# The Bone Marrow-Derived Stromal Cells: Commitment and Regulation of Adipogenesis

**DOI:** 10.3389/fendo.2016.00127

**Published:** 2016-09-21

**Authors:** Michaela Tencerova, Moustapha Kassem

**Affiliations:** ^1^Department of Molecular Endocrinology, Odense University Hospital, University of Southern Denmark, Odense, Denmark; ^2^Danish Diabetes Academy, Novo Nordisk Foundation, Odense, Denmark; ^3^Stem Cell Unit, Department of Anatomy, Faculty of Medicine, King Saud University, Riyadh, Saudi Arabia

**Keywords:** bone marrow stem cells, adipogenesis, secreted factors, bone marrow microenvironment, bone marrow stem cell subpopulations

## Abstract

Bone marrow (BM) microenvironment represents an important compartment of bone that regulates bone homeostasis and the balance between bone formation and bone resorption depending on the physiological needs of the organism. Abnormalities of BM microenvironmental dynamics can lead to metabolic bone diseases. BM stromal cells (also known as skeletal or mesenchymal stem cells) [bone marrow stromal stem cell (BMSC)] are multipotent stem cells located within BM stroma and give rise to osteoblasts and adipocytes. However, cellular and molecular mechanisms of BMSC lineage commitment to adipocytic lineage and regulation of BM adipocyte formation are not fully understood. In this review, we will discuss recent findings pertaining to identification and characterization of adipocyte progenitor cells in BM and the regulation of differentiation into mature adipocytes. We have also emphasized the clinical relevance of these findings.

## Introduction

Bone marrow (BM) is an important compartment of bone, which regulates bone homeostasis. BM can also be perceived as an immune organ as it contains many different cell types secreting a large number of cytokines and immune modulatory factors. Finally, BM is a metabolic organ and has recently been demonstrated to regulate a whole body energy metabolism (1, 2).

The cellular composition of BM is complex as it contains hematopoietic stem cells giving rise to myeloid lineage including osteoclasts and lymphoid lineage giving rise to immune cells. It also contains a stroma compartment containing bone marrow stromal stem cells (BMSC) (also known as skeletal or mesenchymal stem cells) and their differentiated progeny of adipocytes and osteoblasts as well as endothelial cells, pericytes and neuronal cells. The cellular composition of BM changes with age, gender, and metabolic status (3, 4).

**Figure 1:**
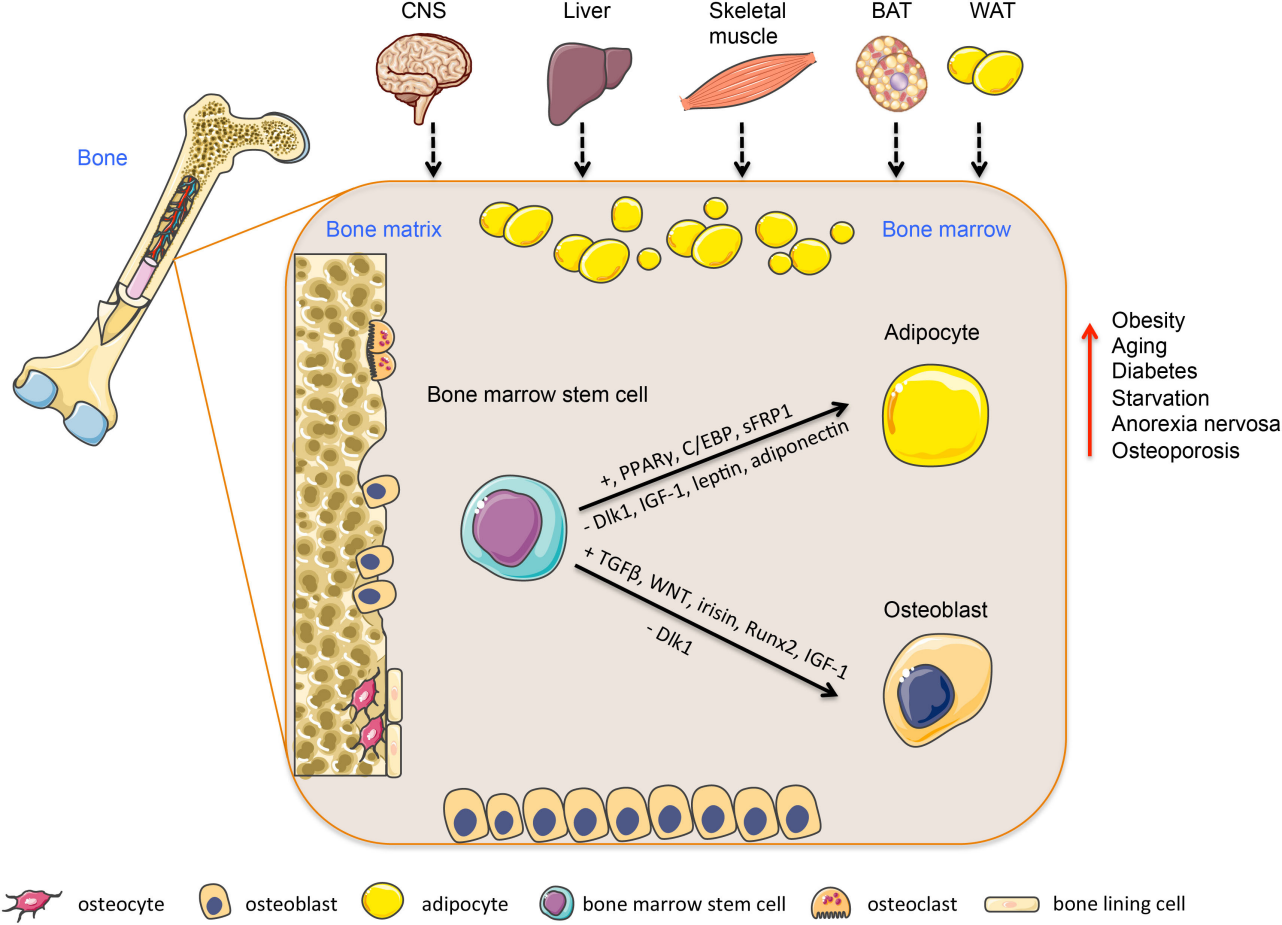
**Regulation of bone marrow stem cells differentiation into adipocytes or osteoblasts**. Bone marrow is a heterogeneous organ, which consists of different cell types participating in bone homeostasis. Among them, most abundant are hematopoietic stem cells (bone resorptive osteoclasts) and mesenchymal stem cells giving rise into bone forming osteoblasts or adipocytes. This process is regulated via several transcription factors and secreted molecules (e.g., PPARs, Wnt, adiponectin, leptin), which are produced locally or released from peripheral tissues, including BAT, WAT, skeletal muscle, liver, or CNS and affecting bone marrow niche through circulation. This multiorgan crosstalk between bone and peripheral tissues plays an important role in the regulation of bone and energy metabolism. Abbreviations: CNS, central nervous system; BAT, brown adipose tissue; WAT, white adipose tissue. Adapted from SERVIER Medical Art; http://www.servier.com/Powerpoint-image-bank

Bone turnover/remodeling is very dynamic and energetically demanding process that consists of two main phases: bone formation mediated by osteoblasts recruited from BMSC and bone resorption mediated by osteoclasts recruited from hematopoietic progenitors. Bone resorption and bone formation are *coupled* in time and space and there is a balance between the amount of bone resorbed by osteoclasts and the amount of bone formed by osteoblasts. These processes of *coupling and balance* are tightly regulated via several factors present in BM microenvironment and also via sympathetic central nervous system (2) (Figure 1). Imbalance between bone resorption and bone formation leads to metabolic bone diseases, including age-related bone loss and osteoporosis.

During the recent years, there has been an increasing interest in understanding the biology of BM adipocyte for a number of reasons. First, it is an abundant cell type in adult BM (5). Second, an increased BM adipose tissue mass has been reported in the conditions of low bone mass, suggesting an abnormal differentiation of BMSC as a possible pathogenetic mechanism to be investigated. Finally, the biological role of BM adipocytes and their differences and similarities with extramedullary adipocytes are not known and may be relevant to bone tissue homeostasis.

In this review, we will present an overview of the BM adipocyte differentiation and its regulation by a number of factors. We will also outline a number of specific signaling pathways that regulate BMSC lineage commitment to adipocytes versus osteoblasts and that can be targeted to enhance bone formation and increase bone mass.

## From Bone Marrow Stem Cells to Committed Adipocytic Cells in the Bone Marrow

*In vitro*, BMSC are plastic-adherent cells, present within BM stroma and capable of clonal expansion and differentiation to osteoblasts and adipocytes. Currently, human BM adipocytes are thought to differentiate from BMSC as evidenced by a large body of both *in vitro* and *in vivo* studies (5). In mice, recent lineage tracing studies employing genetically modified mice, provided evidence for the common stem cell hypothesis for the presence of a common stem cells for osteoblastic and adipocytic cells (6, 7). Table 1 summaries the main characteristics of recently reported BMSC and progenitor cells identified and characterized based on lineage tracing studies employing expression of a number of markers.

**Table 1:** **List of different skeletal progenitor cells in the bone marrow identified by specific cell surface markers and mediators**.

Marker	Function	Differentiation potential	Reference
Nestin	A type VI intermediate filament protein	HSC maintenance	(8)
Gremlin	Inhibitor of BMP in TGF beta signaling pathway	Osteoblast, chondrocyte, reticular cell	(9)
RANKL	Receptor activator of NFκB ligand	Adipocyte	(10)
LepR	Leptin receptor	Adipocyte	(11)
Sox9	Transcription factor	Chondrocyte	(12)
Col2	Type II collagen	Chondrocyte	(13)
CD146	Cell adhesion molecule	Osteoblast	(14)
CD34	Cell adhesion molecule	Osteoblast	(15)

Single cell clonal analysis of cultured BM stromal cells revealed, in addition to BMSC, the presence of independent committed precursors for osteoblasts and adipocytes. Our study of Post et al. demonstrated the existence of these precursors within the murine BM stroma (16). Based on clonal selection, we were able to isolate and characterize the phenotype of two different precursor cell lines isolated from BM of 2- to 3-month-old mice: one cell line is committed to adipocyte differentiation and termed ^adipo^MSC and another one committed to osteoblast and chondrocyte differentiation and termed ^bone^MSC. The two cell lines exhibit distinct morphology and molecular signature. Based on the differentiation marker expression between these two cell lines, we have recently reported that CD34 is differentially expressed in ^bone^MSC and not in ^adipo^MSC and that it can be used to prospective isolation of osteoblastic committed BMSC (15). Examples of studies using lineage tracing to identify committed precursor cells include the study of Yue et al. (11) that employed leptin receptor (LepR) to identify precursor cells with adipocyte differentiation potential. Mice with conditionally deleted LepR in limb bones enhanced osteogenesis and improved fracture healing (11). Another example is the study of Holt et al. (10) that demonstrated the presence of RANKL + preadipocytes in aged mice BM that support osteoclastogenesis. Similar findings have been confirmed in human BM (10, 17). Interestingly, some lineage tracing studies show heterogeneity of BMSC populations with respect to their differentiation potential. Gremlin 1 (Grem1) is a secreted BMP inhibitor and involved in the regulation of adipogenesis in peripheral adipose acting via PPARγ (18). Recent study of Worthley et al. identified Gremlin 1 positive precursor cells that can differentiate into osteoblasts, chondrocytes, and reticular cells, but not to adipocytes (9). All these studies demonstrate the presence of complex cellular heterogeneity within the BM microenvironment and the presence of different committed BMSC subpopulations with a different differentiation potential, whose functions in the BM can be modulated independently during aging and metabolic diseases. It is also possible that these precursor cells number and functions are modulated by physiological conditions and energy demand. Since most of the above-mentioned studies were performed in mice, it is important to define the relevance of these findings to normal human physiology.

## Bone Marrow Adipocytes Versus Extramedullary Adipocytes

Biological differences between BM medullary adipocytes and extramedullary adipocytes are not completely delineated. Several investigators have reported similarities between stromal cells isolated from the BM and subcutaneous stromal cells with respect to molecular phenotype and adipocyte differentiation capacity (19–22). However, significant differences have been observed in their responsiveness to adipocyte differentiation signals, as subcutaneous adipose tissue-derived stem cells (ASC) are better to differentiate to adipocytes compared to BMSC. BM and extramedullary adipocytes are different in cell size, expression of stem cell markers (e.g., Sox2, Nanog, Klf4), presence of extracellular matrix, free fatty acid content, proportion of immune cells in contact with adipocytes, levels of cytokine, and adipokine expression (23–28). Also BMSC have higher expression of inflammatory genes compared to ASC (29). Interestingly, the molecular signature analysis reveals that stromal cells from different tissue compartment are imprinted by their tissue of origin and ASC are enriched in adipogenesis-associated genes compared to MSC from other tissue compartments (19, 30–35). Other differences on cellular and molecular level have been reported (see listed in Table 2).

**Table 2:** **Cellular and molecular characteristics of bone marrow and extramedullary adipocytes**.

Parameters	Bone marrow adipocytes	Extramedullary adipocytes	Reference
Adipocyte size	+	++	(24)
Content of free fatty acids	+	++	(28)
Cytokine expression	↑	↓	(31)
Adipokine expression	↓	↑	(27)
Stem cell markers expression	↓	↑	(26)
Immunomodulatory properties	↑	↓	(31)

A recent paper of Liaw et al. has reported significant differences in lipid composition among different adipocytic cell lines derived from a variety of sources, including white and brown adipose tissue in mice and in adipocytes differentiated from 3T3-L1 cell line and ear mesenchymal cells (36). Unfortunately, the authors did not include BM adipocytes in their studies. This data highlight the differences in lipid metabolism in different adipocyte compartments, which may be mediated by exposure to different bioactive molecules in their microenvironment.

## Bone Marrow Stromal Stem Cell Commitment to Adipocytic Lineage and Regulatory Factors

Bone marrow stromal stem cell commitment to adipocytic lineage is a complex process, which is tightly controlled via several positive and negative regulatory factors activated possibly in a sequential cascade. These factors include steroid hormones, secreted cytokines/adipokines, and transcription factors. Most of these signaling molecules are known for their regulation of adipogenesis in bona fide ASC. Table 3 summarizes the effects of selected regulatory factors on adipocyte differentiation in BMSC and ASC that are discussed in the following paragraphs.

**Table 3:** **List of selected regulatory factors for adipocyte differentiation in bone marrow and adipose-derived stem cells**.

Gene name	Gene symbol	Category	Bone marrow-derived stem cells (BMSC)	Adipose-derived stem cells (ASC)	Reference
Peroxisome proliferated-activated receptor γ	PPARγ	Transcription factor	↑	↑	(37, 38)
CAAT enhancer binding protein	C/EBPα/β	Transcription factor	↑↓	↑	(37–39)
Adiponectin	Adipoq	Soluble mediator	↓	↓	(40–42)
Leptin	Lep	Soluble mediator	↓	↓	(43–45)
Secreted frizzled-related protein 1	sFRP1	Soluble mediator	↑	↑	(46, 47)
Delta like-1/preadipocyte factor 1	Dlk1/Pref-1	Soluble mediator	↓	↓	(48)
Low-density lipoprotein receptor-related protein 5	LRP5	Soluble mediator	↓	↑	(49–51)
Bone morphogenic proteins	BMPs (2,4,7)	Soluble mediator	↓↑ (depends on concentration and differentiation cocktail)	↑ (white and beige)	(52–54)
Insulin growth-like factor 1	IGF-1	Soluble mediator	↓	↑	(55, 56)
Irisin, fibronectin type III domain-containing 5	Fndc5	Soluble mediator	↓	↑ (beige)	(57–59)
Fibroblast growth factor 21	FGF-21	Soluble mediator	↑	↑	(60, 61)
Transforming growth factor beta	TGFβ	Soluble mediator	↓	↓	(62, 63)
Interleukin 1	IL1	Soluble mediator	↓	↓	(62)
Interleukin 6	IL6	Soluble mediator	↓	↓	(62)
Tumor necrosis factor α	TNFα	Soluble mediator	↓	↓	(62)
Heme-oxygenase 1	HO-1	Soluble mediator	↓	↓	(64, 65)

The most characterized transcription factor and a key regulator of adipogenesis is peroxisome proliferated-activated receptor gamma (PPARγ) (37, 66). PPARγ belongs to a nuclear receptor superfamily, which is activated by lipophilic ligands. The activation of PPARγ is necessary and sufficient for adipocyte differentiation and also required for maintenance of differentiated state in BMSC and ASC (67–69). Inhibition of PPARγ *in vitro* impairs adipogenesis, while enhancing osteoblast differentiation in BMSC (67). In mice PPARγ deficiency leads to impaired development of adipose tissue when fed a high-fat diet (HFD) (70). PPARγ is also a target for insulin sensitizing drugs, such as thiazolidinediones in diabetes. However, their use for diabetic patients is associated with a decreased bone mass and increases a risk for fracture. The role of PPARγ activation in age-related increase of BM adipogenesis and decreased osteoblastogenesis has been discussed previously [for more information, see the reviews: Ref. (3, 38, 68, 71)].

Additional transcription factors involved in the regulation of adipogenesis are members of CAAT enhancer binding proteins (C/EBP) family: C/EBPα, C/EBPβ, C/EBPγ and C/EBPδ. Based on the studies performed in 3T3 cell line, C/EBP activation during adipocyte differentiation is synchronized in a temporal manner where early activation of C/EBPβ and C/EBPδ leads to induction of C/EBPα. In BMSC, the function and activation of individual transcription factors exhibited a different pattern (72). Moreover, it has been shown that an isoform of C/EBPβ, liver-enriched inhibitory protein (LIP), which lacks transcriptional binding domain, induces activation of Runx2 and promotes osteogenesis in BMSC (39). C/EBPs crosstalk with PPARγ and regulate each other via a feedback loop (38, 68). C/EBP deficient mice exhibited impaired adipogenesis and insulin sensitivity (73–75). Moreover, C/EBPβ-deficient mice displayed reduced bone mineral density with decreased trabecular number (76, 77). These findings confirm an important role of C/EBPs in the early stage of MSC differentiation and their commitment (78).

The PPARγ-regulated adipokines: leptin and adiponectin are primarily secreted by adipocytes and can regulate adipogenesis (79, 80). *In vitro* leptin inhibits adipogenesis and enhances osteoblastogenesis in human stromal marrow cells (43). On the other hand, leptin-deficient mice *ob/ob* and LepR-deficient *db/db* mice exhibit an increased BM adiposity and low bone mass (79). Leptin regulates bone mass negatively indirectly via sympathetic nervous system (44). Interestingly, selective inhibition of LepR in osteoblastic cells has no effects on bone mass, whereas hypothalamic deletion of LepR leads to a phenotype similar to that of *ob/ob* mice (81), suggesting that the main effects of leptin on bone are centrally mediated. In extramedullary adipocytes leptin impairs adipocyte function (e.g., insulin responsiveness and lipid metabolism) and inhibits lipogenesis (45). In addition, Aprath-Husmann et al. reported no effect of leptin on adipocyte differentiation in ASC of lean and obese subjects (82). Also, leptin levels increase with obesity and diabetes, diseases associated with bone fragility. Thus, leptin seems to exert multiple functions with direct and indirect effects on BM adipocytes and extramedullary adipocytes.

Adiponectin is an adipocyte-secreted factor with insulin sensitizing and anti-inflammatory effects. Adiponectin blocks adipocyte differentiation of BMSC and ASC suggesting an autocrine or paracrine negative feedback loop (40). *In vitro* adiponectin enhances osteoblast differentiation, increases osteoblast proliferation and maturation via cyclooxygenase 2 (Cox2)-dependent mechanism, and inhibits osteoclastogenesis (41, 83). However, *in vivo* effects on bone mass are more complex. Adiponectin regulates bone formation via opposite central and peripheral mechanisms through FoxO1 transcriptional factor. Adiponectin-deficient mice exhibit increased bone mass in young age but low bone mass during aging. This effect is explained by local inhibition of osteoblast proliferation and enhanced osteoblast apoptosis. During aging, this effect is antagonized by adiponectin-mediated effects on hypothalamic neurons that lead to decreased sympathetic tone and, consequently, increased bone mass and decreased energy expenditure (80). Overexpression of adiponectin in adipose tissue causes impairment of adipogenesis and increased preadipocyte factor 1 (Pref-1) expression, which inhibits adipogenesis in mice (42).

In obesity and type 2 diabetes, circulating levels of leptin and adiponectin are differentially regulated (up- and downregulated, respectively). However, their role in the regulation of BM adipogenesis has not been determined. Recently, Yue et al. demonstrated LepR signaling in BMSC promotes adipogenesis and inhibits osteoblastogenesis in response to diet (11). By contrast, activation of adiponectin receptor R1 (AdipR1) in osteoblasts results in enhanced bone formation via GSK-3β/β-Catenin signaling (84). AdipoR1 deficient mice exhibit impaired osteoblast differentiation. The study of Yu et al. highlighted the importance of adiponectin signaling in BMSC mobilization and recruitment during bone fracture repair via increased secretion of stromal cell-derived factor 1 (SDF-1) in mice (85). Cawthorn et al. recently reported increased secretion of adiponectin from BM adipocytes in caloric restriction state that can contribute to its circulating levels (86), suggesting an important endocrine role of BM adipocytes in the regulation of whole body energy metabolism. However, this observation needs further investigation.

Novel factors, which have been identified in our laboratory based on proteomic analysis of secreted factors by committed BM adipocytic cells (^adipo^MSC) and committed BM osteoblastic cells (^bone^MSC), are secreted frizzled-related protein 1 (sFRP1) and Delta-like 1, also known as preadipocyte factor 1 (Dlk1/Pref-1) (16).

Secreted frizzled-related protein 1 is an inhibitor of Wnt signaling that sequester Wnts from their receptors. *In vitro* it inhibits osteoblastogenesis and promotes adipogensis of BMSC by blocking the Wnt signaling (46) *In vivo* sFRP1 inhibited bone formation. Similar effects of sFRP1 have been reported in preadipocytes and primary adipose tissue-derived cells (87, 88).

Delta-like 1/preadipocyte factor 1 is a transmembrane protein, which belongs to a family of epidermal-growth-factor-like repeats containing proteins. Its extracellular domain is proteolytically cleaved by ADAM17/TACE and released as soluble factor circulating in body fluids include amniotic fluid and, hence, its name fetal antigen A (FA1) (89–91). Pref-1 is highly expressed in preadipocytes and its expression decreases during differentiation. Pref-1 overexpression in 3T3-L1 cells blocks adipogenesis (92). Pref-1 regulates adipocyte differentiation via FOXA2 (93), KLF6 (94), and KLF2 (95). Our group has reported that overexpression of Dlk1 in human BMSC inhibits adipocyte and osteoblastic differentiation (48). Interestingly, Dlk1/Pref-1 inhibited differentiation of MSC downstream of C/EBPβ during adipocytic differentiation and Cbfa1/Runx2 during osteoblastic differentiation, suggesting that Dlk1/Pref-1 maintains MSC in a progenitor state. Importantly, we showed a negative effect of soluble FA1 on bone formation in *ex vivo* neonatal calvaria organ cultures. Also transgenic mice with Dlk1 overexpression had reduced fat and bone mass (96). We have also recently reported that FA1 acts as a link between bone and whole body energy metabolism and it interacts with osteocalcin (97).

Additional negative regulators of adipocyte differentiation of BMSC include molecules in Wnt signaling pathway that consists of several ligands, receptors, co-receptors and transcriptional mediators, e.g., β-catenin, which blocks PPARγ and its downstream-regulated genes (98, 99).

Low-density lipoprotein receptor-related protein 5 (LRP5) is a Wnt co-receptor and is involved in activation of canonical Wnt signaling (49, 50, 100). It has been shown to inhibit adipogenesis and promote osteoblastogenesis in BMSC. A gain of function mutation in Lrp5, a clinical condition known as a high-bone-mass phenotype, leads to inhibition of adipogenesis and enhances osteoblastogenesis, which is associated with increased bone mass. On the other hand, Lrp5 loss of function mutation causes severe osteoporosis in mice and humans (49, 50, 100, 101). Recent paper of Loh et al. (49) demonstrated enhanced adipogenesis in lower body fat, e.g., gluteal adipose tissue in high-bone-mass phenotype patients. This finding confirms the results of Palsgaard et al., who reported impaired adipogenesis in LRP5-deficient preadipocytes due to an interaction between LRP5 and insulin receptor (51).

Bone morphogenetic proteins (BMPs) are members of the transforming growth factor β (TGFβ) superfamily that were originally identified based on their ability to induce ectopic bone formation. BMPs have pleiotropic developmental actions, important for stem cell self-renewal and lineage commitment and differentiation (102). BMP4 promotes adipogenesis in peripheral adipose tissue progenitors by increasing transcription activity of PPARγ (18, 103). However, dependent on BMP concentration and receptor activation, they exert different lineage differentiation effects (104). Low concentrations of BMP-2 and BMP-7 induce adipocytic differentiation, whereas high concentrations promote differentiation toward chondrocytes and osteoblasts (105, 106). BMP signaling through type IB BMP receptor (BMPR-IB) plays a crucial role in mediating osteoblast differentiation of BMSC by a Dlx5/Runx2-mediated pathway, while activation of the type IA BMP receptor (BMPR-IA) in BMSC induces PPARs expression and promotes adipocyte differentiation (107, 108).

Another signaling pathway involved in the regulation of adipogenesis is insulin/insulin-like growth factor (IGF-1) pathway (55, 56, 109). IGF-1 has pleiotropic functions in several tissues with regulatory effects on cell proliferation and cell differentiation. It is the most abundant growth factor in the bone matrix. Deletion of IGF-1 in osteocytes caused impaired developmental bone growth in mice (110). IGF-1-osteoblast deficient animals exhibit impaired bone formation and reduction in bone mass (111). Mice treated with recombinant IGF-1 exhibited enhanced bone formation and osteogenesis via activation of mTOR signaling (112). In adipose tissue, IGF-1 plays an important role in adipocyte differentiation, especially in lineage commitment stage where insulin acts predominantly through IGF-1 receptors, which are highly expressed in preadipocytes compared to insulin receptors (113). Blocking downstream molecules in insulin signaling pathway inhibits adipogenesis in preadipocytes (56). Importantly, IGF-1 is also involved in the regulation of energy metabolism and glucose uptake in insulin responsive cells. Therefore, IGF-1 exerts multiple functions dependent on cellular energy needs. However, its role in the regulation of energy metabolism in BMSC and BM adipocytes is not known.

Muscle-secreted proteins, known as myokines, represent a group of regulatory molecules with expanding role in crosstalk between muscle and different organs in the body and with regulatory functions on bone and energy metabolism.

Irisin is a newly identified myokine released from skeletal muscle during exercise through peroxisome proliferator-activated receptor gamma coactivator 1 (PGC1) activation that mediates expression of membrane protein Fibronectin type III domain-containing protein 5 (FNDC5). This is a precursor molecule for irisin, which is subsequently cleaved as the myokine (114). Irisin has a browning effect on white adipose tissue via upregulating uncoupling protein 1 (UCP1) through p38 MAPK and ERK (57, 114). Moreover, recent studies demonstrated that irisin positively affects skeletal system, i.e., it enhances osteoblastogenesis *in vitro* and *in vivo* (58, 59).

Fibroblast growth factor 21 (FGF21) has been identified as a circulating hepatokine with effects on glucose and lipid metabolism. FGF21 is also secreted from adipose tissue and skeletal muscle (115). It exerts a positive effect on adipogenesis via activation of PPARγ in BMSC and adipose tissue progenitors. FGF21-deficient mice exhibit high bone mass and decreased fat formation. Reciprocally, mice overexpressing FGF21 exhibit reduced bone mass (60, 61). In older men, high serum levels of FGF21 are associated with low bone mass (116).

Inflammatory cytokines are mostly produced by immune cells and play an important role in the regulation of bone remodeling and adipocyte formation.

Transforming growth factor beta is a cytokine of the TGF superfamily that promotes preadipocyte proliferation and inhibits adipocyte differentiation. Overexpression of TGFβ in mice leads to impaired adipose tissue development. On the other hand, TGFβ-deficient mice display impaired bone growth and mineralization (117). TGFβ mediates its inhibitory function on adipogenesis via SMAD3, which acts on C/EBPα (56, 118).

The effects of the pro-inflammatory cytokines: interleukin 1 (IL1), interleukin 6 (IL6), and tumor necrosis factor alpha (TNFα) on preadipocytes and BMSC are similar (62). They inhibit adipogenesis by reducing PPARγ and C/EBPα expression and by blocking insulin action via decreasing Glut4 expression in preadipocytes (119). TNFα and IL1 suppress adipocyte differentiation by activation of the TAK1/TAB1/NIK cascade, which in turn inhibits PPARγ activity (120). IL1 and TNFα inhibit adipocyte cell proliferation by activation of several distinct intracellular signaling pathways (e.g., JNK, p38 MAPK) (119, 121, 122). Moreover, IL6 maintains BMSC in undifferentiated state through ERK1/2-mediated mechanism during bone fracture healing (123). On the other hand IL6 enhances osteoblast differentiation of BMSC by decreasing Sox2 expression (124). In estrogen-deficient mouse model, IL6-deficient mice are protected from ovariectomy-induced bone loss (125), suggesting a role in mediating estrogen-deficiency-related bone loss (125).

Heme-oxigenase 1 (HO-1) is a rate-limiting enzyme with anti-inflammatory properties, activated by oxidative stress, which was reported to regulate commitment of human BMSC differentiation to osteoblastic cells. HO-1 acts as an inhibitor of adipogenesis by enhancing Wnt signaling (64). Similar effects were observed in ASC of obese mice, which were treated with HO-1 inducer that led to decreased adiposity in peripheral adipose and BM along with a positive effect on insulin sensitivity (65).

Taken together, the above-mentioned regulatory factors share similar signaling pathways for the regulation of adipocyte differentiation in BMSC and ASC. However, some of these factors, e.g., BMP, IGF-1, and LRP5 display different effects depending on the origin of MSCs, suggesting an important role of local microenvironment. These findings are relevant to the design of potential drugs for targeting BMSC in the context of regulation of bone and energy metabolism. Figure 1 summarizes factors regulating BMSC differentiation and their associated signaling pathways.

## Micro RNA and Regulating Genetic Networks in Adipocytic Differentiation

Micro RNAs (miRNA) are evolutionary conserved short non-coding RNA molecules (containing about 22 nucleotides) that function in RNA silencing and post-transcriptional regulation of gene expression. Accumulating evidence suggests that miRNA regulate fate decisions of stem cells, including self-renewal and differentiation (126, 127).

Several groups have employed global miRNA gene expression profiling during differentiation of human BMSC to identify several miRNAs that regulate BMSC fate and that act as a molecular switch to control adipocyte and osteoblast differentiation fate. Most of miRNAs regulate gene expression of key molecules involved in BMSC differentiation, such as PPARγ, C/EBP, Runx2, Wnt/β-catenin, Lrp5/6, and so on. For example, recent study of Hamam et al. identified miR320 family, whose upregulation in human BMSC enhanced adipocyte differentiation. The biologically relevant gene targets for miR-320c are RUNX2, MIB1 (mindbomb E3 ubiquitin protein ligase 1), PAX6 (paired box 6), YWHAH, and ZWILCH (128). Other miRNAs that have been reported as regulators of adipogenesis include miR-143, -24, -31, -30c, and -642a-3p. More detailed description of their function in the regulation of BMSC differentiation is summarized in these recently published reviews (127, 129, 130). Thus, targeting different miRNAs represents a potential tool for a molecular therapy to regulate BMSC differentiation fate.

## Bone Marrow Adipogenesis in Aging, Osteoporosis, and Metabolic Disorders

*In vivo*, it has been shown an inverse relationship between bone and fat formation in the BM cavity (1, 131, 132). For examples, observed abnormalities of bone remodeling during aging, osteoporosis, estrogen deficiency, chronic glucocorticoid (GC) treatment, immobilization, anorexia nervosa, and Cushing disease are associated with increased adipose tissue accumulation in the BM and decreased bone mass (3, 133–135). One of the cellular explanations that has been put forward to explain this inverse relationship between bone and fat tissue mass in the BM is differentiation reprograming of BMSC toward adipocyte instead of osteoblastic fate (136–138). Moerman et al. reported that aging activates adipogenic and suppresses osteogenic differentiation programs in BMSC in mice (139). Molecular mechanism behind this reprograming machinery is not completely delineated. It has been shown that several extracellular signaling proteins have overlapping roles in BMSC adipogenesis and osteoblastogenesis by modulating the expression and/or activity of adipocyte-specific (e.g., PPARs) or osteoblast-specific (e.g., Runx2 and osterix) transcription factors. Some of these factors play opposing roles in lineage determination, while others function in complementary fashion. Schilling et al. using whole genome analyses identified several genes that could play a role in osteoblast versus adipocyte differentiation of BMSC (138).

Obesity and diabetes are highly prevalent diseases, in which bone mass is also affected (140). Several studies have demonstrated that metabolic complications of diabetes are associated with increased risk for bone fractures. However, it is not clear whether these effects are mediated by changes in BM adipose tissue. Bredella et al. found positive correlation between visceral adipose tissue and BM adiposity as measured in vertebrae of obese premenopausal women. Interestingly, this finding correlates with decreased BMD, even after correcting for the degree of obesity (141). An *in vitro* study reported that incubating BMSC with sera obtained from overweight persons promotes *in vitro* adipocyte differentiation and diminishes osteoblast differentiation (142), suggesting that secreted factors/nutrients present in circulation can affect the differentiation process of BMSC (142). In HFD-induced obesity in mice, an increased osteoclastic bone resorption associated with a lower trabecular bone mass is observed (143). Indeed, saturated fatty acids impair osteoblastogenesis, enhance adipogenesis, and affect cell survival and proliferation of human BMSC (144, 145). Other animal study reported impairment of mitochondrial function and apoptosis of BMSC in obese mice (146).

An increased BM adiposity has been reported in type 1 and type 2 diabetes (T1D and T2D). However, in T1D, there is a decrease in bone mass, whereas T2D is characterized by no change or higher bone mass and paradoxically increased risk for osteoporotic fractures (147–150). Studies are underway to examine the role of impaired glucose metabolism and its associated hyperglycemia and hyperinsulinemia on the biological functions of BMSC. This area of research has also been strengthened by the discovery of osteocalcin as a bone secreted hormone that regulates insulin secretion, proliferation of β-cells, and overall energy metabolism in mice (151, 152) and FA1 as a negative regulator of osteocalcin-induced hypoglycemia (97).

## Targeting Bone Marrow Adipocytes to Increase Bone Mass

Several studies have examined the possibility of reverting the adipocyte differentiation fate of BMSC to bone forming osteoblasts as an approach to increased bone mass during aging and in osteoporosis. A number of molecular studies have investigated therapeutic potential of several factors as regulators of BMSC differentiation fate, which included hormone replacement therapy/small molecules with antagonistic/agonistic effect or neutralizing antibodies.

An example is sclerostin (SOST), which is a glycoprotein produced by osteocytes and acts as an inhibitor of Wnt signaling. SOST inhibits bone formation and increases bone adiposity through possibly targeting differentiation of BMSC (153, 154). Treatment with humanized antibodies against SOST is currently in phase III trials for osteoporosis management (155). Another molecule with a similar function as SOST is Dickkopf-1 (DKK1), which negatively regulates Wnt signaling (156). Growth factors, such as BMPs or activin A, represent other anabolic agents for a potential treatment (157). Recent study of Florio et al. reported promising results on the use of bispecific antibody targeting SOST and DKK1 with an enhanced effect on bone formation in rodents and non-human primates (158). However, further clinical studies are needed to investigate the effectiveness of combined treatment, especially in patients with severe osteoporosis.

Some anti-diabetic drugs designed to improve insulin sensitivity and adipogenesis in the peripheral tissues have unfortunately the side effects on bone mass with increased fracture risk, e.g., thiazolidinediones due to partly enhanced BM adiposity (71, 159, 160). Thus, one of the research goals is to design anti-diabetic drugs with minimal negative effects on bone. Recently used anti-diabetic drugs in the clinic include the incretin-based therapies (GLP-1 receptor agonists, DPP-4 inhibitors) and drugs targeting sodium-glucose co-transporter 2 (SGLT2)-inhibitors. However, their effects on bone mass and fracture risk need to be determined (161).

## Conclusion/Future Perspectives

MSC commitment to differentiate into osteoblasts or adipocytes and, consequently, the balance between bone mass and BM adipose tissue mass is a complex and dynamic process, which is regulated and fine tuned by a large number of bioactive molecules. The sequential cascade of these processes and how they modulate BMSC differentiation is currently under intensive investigation. Future studies need to identify the biological functions of BM adipocytes not only in relation to bone remodeling but also as part of the overall regulation of energy metabolism. The findings of novel secreted molecules involved in the regulation of cellular fate of BMSC provide new possible anabolic therapies for treating clinical conditions of low bone mass and possibly disturbances in energy metabolism.

## Author Contributions

MT and MK researched data, wrote manuscript and reviewed the final manuscript.

## Conflict of Interest Statement

The authors declare that the research was conducted in the absence of any commercial or financial relationships that could be construed as a potential conflict of interest.
